# Supplementary material for: Map-based experience replay: a memory-efficient solution to catastrophic forgetting in reinforcement learning
Source: Front Neurorobot. 2023 Jun 27;17:1127642. doi: 10.3389/fnbot.2023.1127642 (PMC10333526; doi:10.3389/fnbot.2023.1127642)
Supplement: Supplementary file 1 [file Presentation_1.pdf]

frontiers

Supplementary Material

APPENDIX A: ADDITIONAL EXPERIMENTS

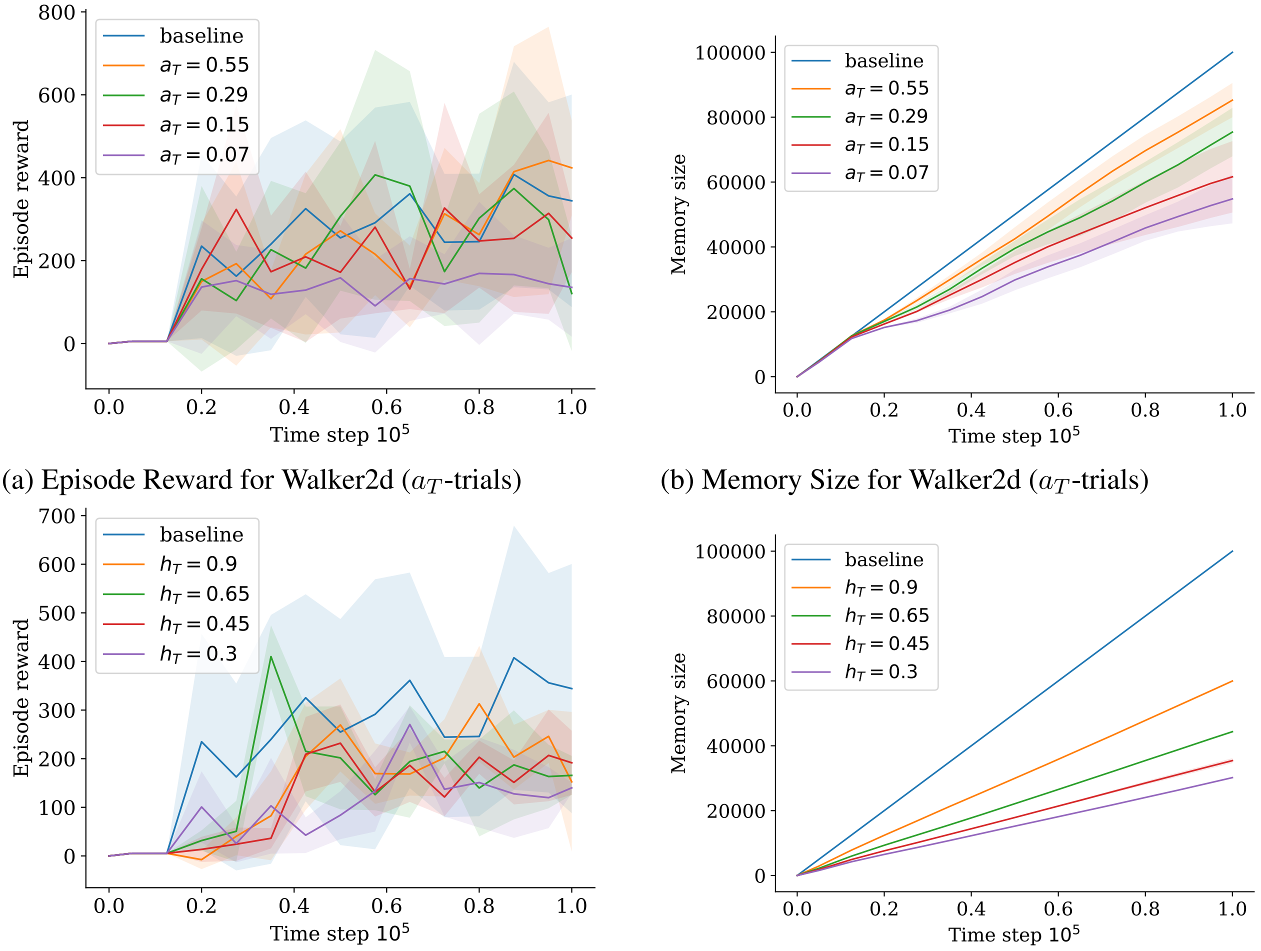

(a) Episode Reward for Walker2d (a_T-trials)

(b) Memory Size for Walker2d (a_T-trials)

(c) Episode Reward for Walker2d (h_T-trials)

(d) Memory Size for Walker2d (h_T-trials)

Figure S1. Episode reward and memory size for different activation (top raw, $h_T = 1$) and habituation (bottom raw, $a_T = 1$) threshold values for Walker2d-v2. Shaded area shows standard deviation.

Additional experiments to support our analysis were done on the MuJoCo environment Walker2d-v2, which we chose as it is similar to HalfCheetah environment in dimensions (state-space dimensions: 17, action-space dimensions: 6). Each trial was run 4 times and then averaged. The trials were run on two NVIDIA GeForce RTX 2080 Ti GPUs.

Although Walker2d has a learning curve much less steep than other environments with trials being relatively close score-wise, Fig. S1(a-d) visibly demonstrates support of our general analysis. Considerable memory reduction down to about 60% still achieve results very comparable to the baseline. As shown in Fig. S2, going from the great performance differences at the 0.6-mark, the superiority of the activation threshold in reducing memory seems to hold up.

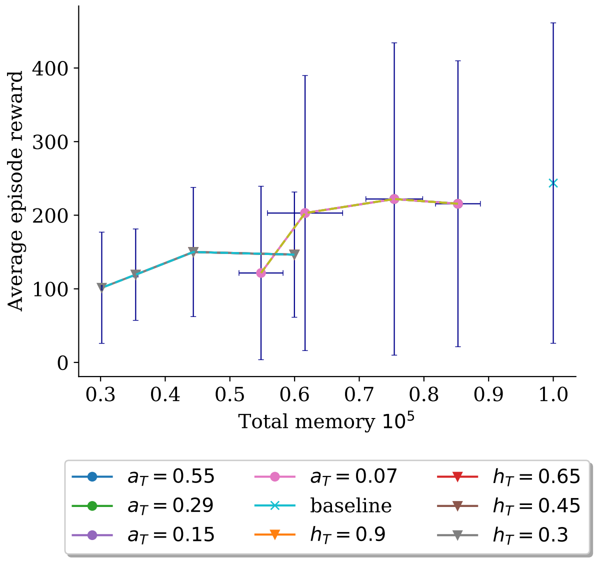

Figure S2. Comparison between the a_T-trial (dots) and the h_T-trial (triangles) of the ratio of average reward over all episodes to the memory size after training. Experiments done on the MuJoCo environment Walker2d. Each run is one dot/triangle; dots/triangles are connected for easier comparison of approximate underlying function. Error bars show mean standard deviation.
